# Optimal Preoperative Multidisciplinary Treatment in Borderline Resectable Pancreatic Cancer

**DOI:** 10.3390/cancers13010036

**Published:** 2020-12-24

**Authors:** Nana Kimura, Suguru Yamada, Hideki Takami, Kenta Murotani, Isaku Yoshioka, Kazuto Shibuya, Fuminori Sonohara, Yui Hoshino, Katsuhisa Hirano, Toru Watanabe, Hayato Baba, Kosuke Mori, Takeshi Miwa, Mitsuro Kanda, Masamichi Hayashi, Koshi Matsui, Tomoyuki Okumura, Yasuhiro Kodera, Tsutomu Fujii

**Affiliations:** 1 Department of Surgery and Science, Faculty of Medicine, Academic Assembly, University of Toyama, Toyama 9300194, Japan; nana@med.u-toyama.ac.jp (N.K.); isaku@med.u-toyama.ac.jp (I.Y.); chopper@med.u-toyama.ac.jp (K.S.); yui@hoshi-co.jp (Y.H.); hrnkths21@yahoo.co.jp (K.H.); toruwatanabetoru@yahoo.co.jp (T.W.); h881088@med.u-toyama.ac.jp (H.B.); mori0824@med.u-toyama.ac.jp (K.M.); tmiwa@med.u-toyama.ac.jp (T.M.); kmatsui@med.u-toyama.ac.jp (K.M.); okumura@med.u-toyama.ac.jp (T.O.); 2 Department of Gastroenterological Surgery (Surgery II), Nagoya University Graduate School of Medicine, Nagoya 4668550, Aichi, Japan; suguru@med.nagoya-u.ac.jp (S.Y.); takamihideki@med.nagoya-u.ac.jp (H.T.); fsonohara@med.nagoya-u.ac.jp (F.S.); m-kanda@med.nagoya-u.ac.jp (M.K.); m-hayashi@med.nagoya-u.ac.jp (M.H.); ykodera@med.nagoya-u.ac.jp (Y.K.); 3 Biostatistics Center, Graduate School of Medicine, Kurume University, Kurume, Fukuoka 8300011, Japan; kmurotani@med.kurume-u.ac.jp

**Keywords:** pancreatic cancer, borderline resectable, neoadjuvant treatment, chemoradiotherapy, prognostic nutritional index

## Abstract

**Simple Summary:**

For borderline pancreatic cancer, upfront surgery was standard in the past, and the usefulness of neoadjuvant treatment has been reported in recent years. However, few studies have been conducted to date on whether there is a difference in optimal treatment between borderline resectable pancreatic cancer invading the portal vein (BR-PV) or abutting major arteries (BR-A). The objective of this study was to investigate the optimal neoadjuvant therapy for BR-PV or BR-A. We retrospectively analyzed 88 patients with BR-PV and 111 patients with BR-A. In this study, we found that neoadjuvant treatment using new chemotherapy (FOLFIRINOX or gemcitabine along with nab-paclitaxel) is essential for improving the prognosis of BR pancreatic cancer. These findings suggest that prognosis may be prolonged by maintaining good nutritional status during preoperative treatment.

**Abstract:**

*Background:* The objective of this study was to investigate the optimal neoadjuvant therapy (NAT) for borderline resectable pancreatic cancer invading the portal vein (BR-PV) or abutting major arteries (BR-A). *Methods:* We retrospectively analyzed 88 patients with BR-PV and 111 patients with BR-A. *Results:* In BR-PV patients who underwent upfront surgery (*n* = 46)/NAT (*n* = 42), survival was significantly better in the NAT group (3-year overall survival (OS): 5.8%/35.5%, *p* = 0.004). In BR-A patients who underwent upfront surgery (*n* = 48)/NAT (*n* = 63), survival was also significantly better in the NAT group (3-year OS:15.5%/41.7%, *p* < 0.001). The prognosis tended to be better in patients who received newer chemotherapeutic regimens, such as FOLFIRINOX and gemcitabine with nab-paclitaxel. In 36 BR-PV patients who underwent surgery after NAT, univariate analysis revealed that normalization of tumor marker (TM) levels (*p* = 0.028) and preoperative high prognostic nutritional index (PNI) (*p* = 0.022) were significantly associated with a favorable prognosis. In 39 BR-A patients who underwent surgery after NAT, multivariate analysis revealed that preoperative PNI > 42.5 was an independent prognostic factor (HR: 0.15, *p* = 0.014). *Conclusions:* NAT using newer chemotherapy is essential for improving the prognosis of BR pancreatic cancer. These findings suggest that prognosis may be prolonged by maintaining good nutritional status during preoperative treatment.

## 1. Introduction

Despite considerable improvements in diagnostic and therapeutic options, pancreatic ductal adenocarcinoma (PDAC) mostly remains a fatal disease worldwide [1]. Radical resection without residual tumor remains the only established curative treatment for PDAC. However, much more intervention is required beyond resection alone. A simple explanation for the poor consequences after resection is that almost every patient has microscopic disease remaining [2]. When a patient is diagnosed with PDAC and the optimal treatment strategy is considered, it is common to make a decision based on the resectable classification rather than the stage classification. The National Comprehensive Cancer Network (NCCN), an alliance of 25 cancer centers in the United States, have proposed a resectable classification for pancreatic cancer [3]. However, the NCCN guidelines are revised and updated annually and are considered to be very complex; thus, utilizing the NCCN classification system for resectability in clinical practice is difficult. Therefore, the Japan Pancreas Society (JPS) proposed novel and simplified resectability criteria in 2016 [4] based on the most recent NCCN guidelines [3].

The JPS published the 7th edition of the Classification of Pancreatic Carcinoma, and a unique resectable classification for borderline resectable (BR) was proposed (BR-A: BR-PDAC due to the infiltration of celiac and/or superior mesentery arteries, BR-PV: due only to the infiltration of the portal system). BR pancreatic cancer is a distinct subset of locally advanced pancreatic cancer first identified by Varadhachary et al. in 2006 [5]. It was hoped that the BR group would represent a subset of pancreatic cancer whose outcomes might be intermediate between the outcomes of patients with radiologically and technically resectable (R) and unresectable (UR) disease. With currently available operative techniques, patients with BR cancer are at high risk for margin-positive resection [6]. Therefore, the criteria for resectability are clinically important for determining the need for preoperative (neoadjuvant) systemic therapy and/or local-regional chemoradiation to maximize the potential for R0 resection and to avoid R2 resection [7]. For BR pancreatic cancer, upfront surgery was standard in the past, and the usefulness of neoadjuvant treatment (NAT) has been reported in recent years [8,9,10]. However, few studies have been conducted to date on whether there is a difference in optimal treatment between BR-PV and BR-A.

The objective of this study was to investigate the optimal preoperative multidisciplinary treatment for BR pancreatic cancer. Patients who had received treatment for BR-PDAC at two regional high-volume centers were reviewed retrospectively, and we analyzed survival differences among subgroups defined based on this novel classification system of resectability.

## 2. Results

### 2.1. Cohort Outline

**Figure 1:**
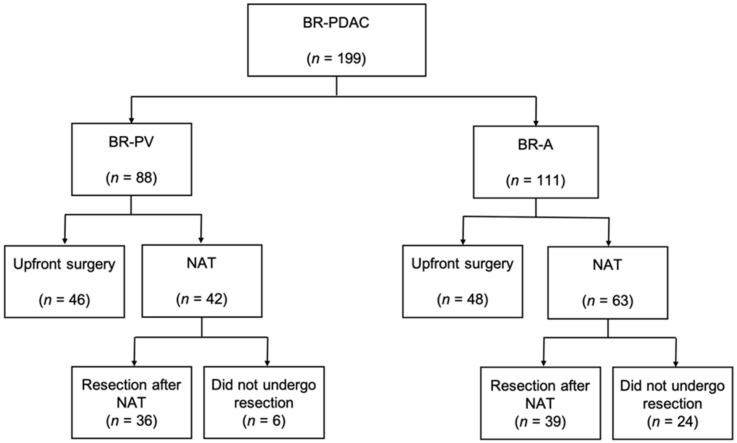
Study profiles and clinical courses of the enrolled patients. BR, borderline resectable; PDAC, pancreatic ductal adenocarcinoma; NAT, neoadjuvant treatment.

We identified 199 patients who were diagnosed with BR-PDAC (Figure 1). Among them, 88 patients were diagnosed with BR-PV PDAC, and 111 patients were diagnosed with BR-A PDAC.

Of 88 BR-PV patients, 46 patients underwent upfront surgery, and 36 patients underwent resection after NAT. The other 6 patients did not undergo surgery because of chemotherapeutic failure or best supportive care. Of 111 BR-A patients, 48 patients underwent upfront surgery, and 39 patients underwent resection after NAT. The other 24 patients did not undergo surgery because of chemotherapeutic failure or best supportive care.

### 2.2. The Clinical Characteristics of BR-PDAC Patients

For patients who were enrolled in this study, detailed cohort demographics are summarized in Table 1. The median age was 66 years in BR-PV patients and 67 years in BR-A patients. Preoperative image examination revealed that the location of the tumor was dominant (BR-PV: 95%, BR-A: 75%) on the head side in both BR-PV and BR-A; thus, pancreatic head resection tended to be more frequent (BR-PV: 84%, BR-A: 60%).

In the BR-PV patients, 26 (30%) patients were treated with newer chemotherapeutic regimens such as FOLFIRINOX (FFX) and gemcitabine along with nab-paclitaxel (GnP). The median length of therapy was 2.1 months. In the BR-A patients, 36 (32%) patients were treated with newer chemotherapeutic regimens with a median length of 2.7 months.

The median baseline CA19-9 level at diagnosis was higher than the median at surgery. Additionally, both the BR-PV and BR-A groups had lower median CA19-9 levels at operation in patients who underwent surgery after NAT than in those who underwent upfront surgery. In both BR-PV and BR-A patients, approximately 20% of patients had a ≥90% decrease in CA19-9 levels compared to that before NAT. This suggests that preoperative NAT may be expected to significantly reduce tumor markers (TMs), as in previous reports [11].

The median baseline nutritional parameters at operation were as follows (in BR-PV/BR-A): controlling nutritional status (CONUT): 2/2, Glasgow prognostic score (GPS): 0/0, modified GPS (mGPS): 0/0, neutrophil/lymphocyte ratio (NLR): 2.4/2.5, platelet/lymphocyte ratio (PLR): 129.2/83.0, prognostic nutritional index (PNI): 46.0/44.5, lymphocyte/monocyte ratio (LMR): 3.6/3.8, systemic immune inflammation index (SII): 380.1/482.8, and C-reactive protein (CRP)/albumin ratio: 0.07/0.03.

For patients with BR-PDAC who were underwent surgery, detailed cohort demographics are summarized in Table 2. The median age was 65 years in BR-PV patients and 67 years in BR-A patients. In operation, venous resection was performed in 72 (88%) patients with BR-PV and 62 (71%) patients with BR-A. Moreover, arterial resection was performed in 5 (6%) patients with BR-PV and 12 (14%) patients with BR-A.

In addition, we compared the backgrounds of patients who underwent upfront surgery and those who underwent NAT. Details are shown in Table 3. For both BR-A and BR-PV, the CA19-9 level at operation was lower in the NAT group. There was no significant difference in preoperative nutritional status.

### 2.3. Comparison of Prognosis of Upfront Surgery vs. Neoadjuvant Treatment by Intention to Treat Analysis

**Figure 2:**
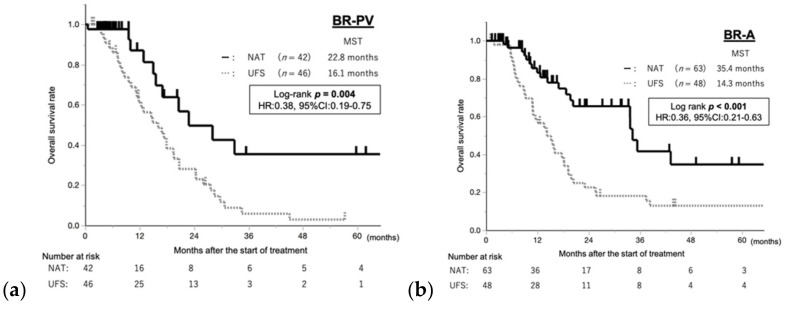
The overall survival in comparison between patients treated with and without NAT in the (**a**) BR-PV and (**b**) BR-A groups. The prognosis of patients treated with NAT was significantly better than that of patients treated without NAT in the BR-PV and BR-A groups (*p* = 0.004 and *p* < 0.001). NAT, neoadjuvant treatment; UFS, upfront surgery; MST, median survival time; HR, hazard ratio; CI, confidence interval.

In BR-PV patients who underwent upfront surgery (*n* = 46)/NAT (*n* = 42), survival was significantly better in the NAT group (*p* = 0.004) (Figure 2). In BR-A patients who underwent upfront surgery (*n* = 48)/NAT (*n* = 63), survival was significantly better in the NAT group (*p* < 0.001). This analysis was performed by intention-to-treat analysis.

The 36-month (3-year) OS rates with upfront surgery and NAT were 5.8% versus 35.5% in BR-PV patients and 15.5% versus 41.7% in BR-A patients, respectively.

### 2.4. Comparison of Regimens in Neoadjuvant Treatment Induction Cases

We compared the regimens of neoadjuvant treatment in each group (Table 4).

In BR-PV patients who underwent FFX/GnP (*n* = 26) vs. gemcitabine (GEM)/S-1 (*n* = 2) vs. GEM/S-1 with radiotherapy (RT) (*n* = 14), the median survival times (MSTs) were 32.9, 10.0 and 20.6 months, respectively, and the prognosis tended to be better in the FFX/GnP group. The number of resected cases was 36 (86%).

In BR-A patients who underwent FFX/GnP (*n* = 29) vs. FFX/GnP with RT (*n* = 7) vs. GEM/S-1 (*n* = 10) vs. GEM/S-1 with RT (*n* = 17), the MSTs were 35.4, 18.7, 43.2 and 19.7 months, respectively, with a better prognosis in the FFX/GnP group. The number of resected cases was 39 (62%).

The R0 rate tended to be higher in regimens with RT.

### 2.5. Prognostic Factors in Patients Who Underwent Resection after NAT

#### 2.5.1. Definition of Cutoff Values for PNI

**Figure 3:**
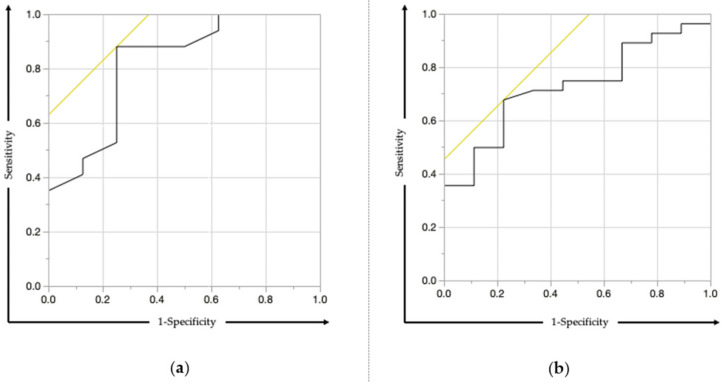
ROC analysis for the prediction of 2-year survival according to the preoperative PNI. (**a**) The AUC was 0.820 in BR-PV patients. (**b**) The AUC was 0.728 in BR-A patients. AUC, area under the curve; PNI, prognostic nutritional index.

Receiver operating characteristic (ROC) curve analysis was performed with data from 36 BR-PV PDAC patients who underwent surgical resection between January 2002 and December 2018 to examine the association between PNI and 2-year survival. The area under the curve (AUC) was 0.728, and the best cutoff value was calculated as 42.65 (Figure 3a). Moreover, ROC curve analysis was performed with data from 39 BR-A PDAC patients. The AUC curve was 0.820, and the best cutoff value was calculated as 42.50 (Figure 3b). We eventually determined that the cutoff value for PNI was 42.5.

#### 2.5.2. Univariate and Multivariate Analyses of Prognostic Factors in BR-PDAC Patients Who Underwent Resection after Neoadjuvant Treatment

Table 5 shows the results of univariate analysis of prognostic factors in BR-PDAC patients who underwent resection after NAT. The cutoff values for continuous variables except preoperative PNI were determined using median values of all BR-PDAC patients who underwent resection after NAT.

In 36 BR-PV patients who underwent surgery after NAT, univariate analysis of overall survival revealed that normalization of TM levels (*p* = 0.028), preoperative GPS = 0 (*p* = 0.025), and preoperative high PNI (*p* = 0.022) were significantly associated with better prognosis. There was no significant difference in the multivariate analysis.

In 39 BR-A patients who underwent surgery after NAT, univariate analysis revealed that normalization of TM levels (*p* = 0.033), preoperative high PNI (*p* = 0.013), and intraoperative blood loss ≤ 830 mL (*p* = 0.013) were significantly associated with better prognosis. Multivariate analysis showed that preoperative PNI > 42.5 was an independent prognostic factor (HR: 0.15, *p* = 0.014). There was no correlation between the length of NAT and additional RT in survival in either BR-PV or BR-A.

#### 2.5.3. Prognosis of BR-PDAC Patients Who Underwent Resection after Neoadjuvant Treatment Based on PNI

**Figure 4:**
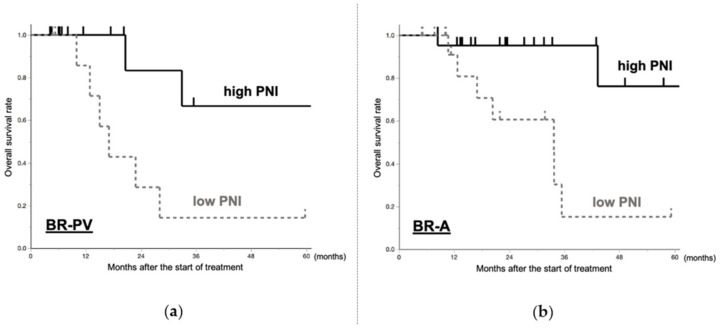
Comparison of the overall survival between high preoperative PNI and low preoperative PNI in the (**a**) BR-PV and (**b**) BR-A groups. The prognosis of patients with high preoperative PNI was significantly better than that of patients with low preoperative PNI in the BR-PV and BR-A patients (*p* = 0.029 and *p* = 0.012). PNI, prognostic nutritional index.

In BR-PV patients who underwent resection after NAT (*n* = 36), survival was significantly better in the high PNI (preoperative PNI > 42.50) group (*p* = 0.029, HR:0.16, 95%CI:0.03–0.83) (Figure 4a). In BR-A patients who underwent resection after NAT (*n* = 39), survival was significantly better in the high PNI (preoperative PNI > 42.50) group (*p* = 0.012, HR:0.13, 95%CI:0.03–0.64) (Figure 4b).

Moreover, comparing the high preoperative PNI and low preoperative PNI, there was no statistically significant difference regarding postoperative complications (Clavien–Dindo grade III or more) in both BR-PV and BR-A patients (*p* = 0.644 and *p* = 0.580, respectively).

## 3. Discussion

There have been many analytical studies on R-PDAC and UR-PDAC, but few have focused on BR-PDAC. The usefulness of NAT for BR-PDAC has been highlighted in several articles [8,9,10,11]. Unfortunately, previous reports often analyzed mixed cohorts of patients, including those with BR and locally advanced UR-PDAC, those with BR-PDAC due to the infiltration of celiac and/or superior mesentery arteries (BR-A) and those with only infiltration of the portal system (BR-PV) [12,13]. The surgical strategy and outcome definitely differ between PDAC abutted to the major arteries and PDAC exclusively involving the PV system [14]. Murakami et al. reported that the BR-PV group had a significantly more favorable overall survival than the BR-A group in an analysis of BR patients who underwent upfront surgery [15]. Thus, it seems inappropriate to discuss the efficacy of the treatment strategy using such admixture.

In the present study, we differentiated between BR-A and BR-PV and analyzed the optimal preoperative multidisciplinary treatment and nutritional status before and after NAT for each type. There have been no comprehensive analyses focusing on surgical strategy for this cohort.

### 3.1. BR-PV

We retrospectively reviewed 88 patients with BR-PV PDAC. The results showed that the prognosis of BR-PV patients who underwent resection after NAT was significantly better than that of patients who underwent upfront surgery without NAT.

Fujii et al. reported that neoadjuvant chemoradiotherapy (NACRT) with S-1 rather than upfront surgery improves R0 rates and increases the survival of patients with BR-PV adenocarcinoma of the pancreatic head but not that of patients with R-PDAC [6]. However, the prognosis tended to be better in the FFX/GnP group than in the NACRT with old chemotherapy group in the present study. Although only 14 BR-PV patients underwent NACRT in this study, chemotherapeutic regimens such as FFX/GnP are expected to be a promising option.

### 3.2. BR-A

We retrospectively reviewed 111 patients with BR-A PDAC. Similar to that of BR-PV patients, the prognosis of BR-A patients who underwent resection after NAT was significantly better than that of those who underwent upfront surgery without NAT. Moreover, in patients with BR-A, the use of NAT with FFX/GnP significantly prolonged the prognosis.

Nagakawa et al. reported that NACRT, which combines chemotherapy with GEM/S-1, with intensity modified radiotherapy (IMRT) had fewer adverse events and improved the prognosis of BR-A [16]. In addition, they also reported that the R0 resection rate after NACRT was 94.7%. In the present study, the R0 resection rate also tended to be higher in patients who underwent additional RT, although additional RT failed to contribute to patient survival.

Hackert et al. reported that resection rates following FFX were 61% compared with 46% after GEM and RT in patients with locally advanced PDAC [17]. This study did not investigate NACRT, which combines new chemotherapy and RT; thus, it cannot be affirmed. However, the combination of radiation with more effective chemotherapy, such as FFX or GnP, is expected to improve the surgical consequences of BR-A patients. On the other hand, effective chemotherapy may lead to more adverse events. There is a report that NAT with FFX followed by IMRT concurrent with fixed-dose-rate GEM in BR-PDAC is feasible and tolerated [18]. Therefore, the IMRT technique may enable the application of NACRT in combination with more effective chemotherapy.

Over the past decade, newer chemotherapeutic regimens, including FFX and GnP, have emerged as new standard therapies for PDAC, which was formerly a lethal disease, and many studies have demonstrated promising survival rates [8,9,10,12,13]. However, there are few prospective randomized controlled studies to confirm the efficacy of NAT for patients with BR-PV and BR-A [16]. Evaluation of NAT is required for patients with BR-PV and BR-A in the setting of prospective trials.

### 3.3. BR-PV and BR-A

From previous reports as well as the results of this study, surgery after NAT is arguably more beneficial than upfront surgery in patients with BR-PDAC; therefore, we focused only on the patients who underwent NAT in the analysis after Section 2.4.

We demonstrated that the long-term survival of patients who underwent resection after NAT was significantly associated with good nutritional status, such as a PNI of more than 42.5 at the time of operation but not at diagnosis. Several studies have reported that preoperative nutrition indices, such as CONUT, mGPS, and PNI, are linked to the prognosis of various malignancies [19,20,21]. In pancreatic cancer, some indices have also been reported to have an independent association with survival in patients with resectable or BR-PDAC after pancreatectomy [22,23]. Moreover, there was a report that NAT for PDAC could aggravate nutritional status and hamper its postoperative recovery and that malnutrition might decrease the tolerance of NAT [24]. While definitive conclusions cannot be drawn from this retrospective study, these results strongly suggest the need for nutritional care during NAT in patients with PDAC. Systemic chemotherapy generally tends to worsen the patient’s nutritional status as a side effect, including loss of appetite or dysgeusia [25]. Active nutritional care during NAC may minimize malnutrition, possibly improving the survival of BR-PDAC patients.

Furthermore, both the BR-PV and BR-A groups had lower median CA19-9 levels at operation in patients who underwent surgery after NAT than those who underwent upfront surgery. Although no significant difference was found in the multivariate analysis, normalization of TM levels was significantly associated with better prognosis in both BR-PV and BR-A patients. Chen et al. reported that long-term (approximately 6 months) chemotherapy after preoperative chemoradiotherapy may improve the prognosis in patients with potentially resectable/BR/UR-PDAC [26]. Satoi et al. also reported that the prognosis was prolonged by giving chemotherapy for 240 days or more in patients with UR-PDAC [27]. Conversely, the results of this study showed that the duration of NAT did not correlate with prognosis for BR-PDAC. It is suggested that the prognosis may be prolonged by surgery after the TM level is greatly reduced, not the length of NAT. Some patients with BR-PDAC became unresectable due to the progression of disease, such as distant metastasis during NAT. Therefore, assessment of TMs may be a more sensitive measure of the indications for resection than the length of treatment. Further exploration will be required for the optimal NAT duration and timing of surgery.

There are several limitations to our study. First, this study was retrospective in design with a relatively small number of patients. Second, the distribution of patients in the different treatment arms was unbalanced. Third, the number of patients receiving NAT has increased since around 2010. The proportion of patients receiving NAT and upfront surgery has changed significantly between 2002 and 2018. Fourth, indications for CRT were biased because CRT was recommended at the physicians’ discretion. Finally, we have not started nutritional support for patients with NAT. We plan to explore the effect of nutritional support during NAT for patients with BR-PDAC to confirm the clinical relevance of this study. Further studies with more patients and longer observation periods are needed to evaluate the optimal and detailed strategy of multidisciplinary treatment for BR-PDAC.

In conclusion, these findings suggest that NAT followed by surgery rather than upfront surgery offers clinical benefits to patients with BR-A PDAC. Moreover, nutritional management during NAT may lead to a better prognosis.

## 4. Materials and Methods

### 4.1. Study Design

A prospectively maintained pancreatic resection database at two regional high-volume centers, Toyama University Hospital (Toyama, Japan) and Nagoya University Hospital (Nagoya, Japan), was queried to identify patients with BR PDAC who started the initial treatment between January 2002 and December 2018. This study conforms to the ethical guidelines of the World Medical Association Declaration of Helsinki-Ethical Principles for Medical Research Involving Human Subject. Written informed consent for inclusion in the study, as required by the institutional review board of both institutions, was obtained from all patients.

We retrospectively examined 199 patients with BR pancreatic cancer (88 patients with BR-PV and 111 patients with BR-A). For BR-PV and BR-A, the following points were investigated:

1. Comparison of prognosis of upfront surgery vs. NAT by intention to treat analysis;
2. Comparison of regimens in patients who underwent NAT;
3. Prognostic factors in patients who underwent resection after NAT.

### 4.2. Definitions of BR-PV PDAC and BR-A PDAC Patients

The preoperative resectability status was categorized into R (resectable), BR-PV, BR-A, UR (unresectable)-LA, and UR-M (metastatic) according to the 7th edition of the JPS classification (Table 6) [4].

Patient eligibility was rigorously defined using thin-slice multidetector-row computed tomography. All images were reviewed by two or more experienced radiologists to reaffirm the preoperative staging. Consequently, 199 patients with BR-PDAC (88 patients with BR-PV and 111 patients with BR-A) were enrolled in this study.

### 4.3. Neoadjuvant Treatment

Some patients received NAT from diagnosis with the following regimens: GnP, FFX, modified FFX (mFFX), or GEM with oral S-1 (the oral 5-fluorouracil prodrug tegafur with oteracil and gimeracil). These chemotherapeutic regimens were selected depending on the patient’s background and the period of enrollment. We performed NAT in 105 patients (42 patients with BR-PV and 63 patients with BR-A) from which informed consent was obtained depending on their condition and tumor status. CRT consisted of a photon/proton external beam with 50.4 Gy delivered in 28 fractions combined with systemic chemotherapy involving oral S-1, which was administered twice daily (80 mg/m^2^/day) from days 1 to 14 and from days 22 to 35.

### 4.4. Postoperative Adjuvant Therapy

Postoperative adjuvant chemotherapy was applied unless contraindicated by the patient’s condition. In short, the patients received GEM or S-1 for 6 months according to the protocol that was available at the time of treatment [28,29]. GEM at a dose of 1000 mg/m^2^ was administered weekly for 3 weeks followed by 1 week of rest; oral S-1 (80 mg/m^2^/day) was administered from days 1 to 28 followed by a 2-week rest period. Chemotherapy was initiated at <2 months after the operation in all patients who were considered eligible for the treatment. Computed tomography was routinely performed every 6 months as a postoperative follow-up imaging examination, and a blood test, including evaluation of TMs, was performed every 2 months to evaluate the recurrent disease.

### 4.5. Data Collection

We collected patient data from the medical records. Pretreatment factors included age, sex, body mass index, tumor size, and blood test results, including serum CA19-9 level. Preoperative factors included chemotherapeutic regimen, length of NAT, and change in CA19-9 level. Perioperative factors included surgical procedures, region of tumor, operative time, blood loss volume, blood transfusion, incidence of postoperative complications according to the Clavien–Dindo classification [30], length of hospital stay, and 90-day mortality.

The tumor-node-metastasis staging system for pancreatic tumors of the seventh edition of the Union for International Cancer Control was applied [31]. The pathological data collected included tumor grade, number of positive lymph nodes, resection margins, perineural invasion, PV invasion, and artery invasion. The surgical margin in this study denoted either the stump of the pancreas or the bile duct or the dissected plane around the pancreas as described by Staley et al. [32]. If viable cancer cells were detected microscopically at the tip of any of these sites, the surgical margin was noted as positive. If the tumor was located at a distance of >1 mm from the surgical margin, the margin was noted as negative.

### 4.6. Nutritional Status

In the current study, we also investigated several nutritional parameters at diagnosis and at operation, such as the GPS [33], mGPS [33], CONUT [19], PNI [22,33], NLR [33,34], PLR [33], LMR [34], and SII [35], to verify their impact on the operative outcome and the prognosis.

### 4.7. Statistical Analysis

A biostatistician (K.M.) was responsible for the statistical analysis. The Kaplan–Meier method was used to calculate survival rates, and the difference in survival curves was analyzed by the log-rank test. To detect prognostic factors for survival, we performed Cox proportional hazard analysis, and hazard ratios and 95% confidence intervals (CIs) were calculated. Goodness-of-fit for preoperative PNI was assessed by calculating the AUC of the ROC curve, and the optimal cutoff value was determined using the Youden index. Other cutoff values in Table 3 used their median values. Differences in nominal data between the two groups were examined using the chi-square test or Fisher’s exact test when the expected value was <5. Differences in quantitative variables were evaluated using Student’s *t*-test or the Mann–Whitney *U* test if the distribution was abnormal. A *p* value < 0.05 was considered statistically significant. All statistical analyses were performed using JMP statistical software (version 14.2; SAS Institute, Cary, NC, USA).

## 5. Conclusions

NAT using chemotherapy such as FFX or GnP is essential for improving the prognosis of BR pancreatic cancer. This suggests that prognosis may be improved by maintaining good nutritional status during preoperative treatment, not by the length of preoperative treatment. In addition, normalization of TMs by preoperative treatment contributes to the prolongation of survival.

## Figures and Tables

**Table 1:** Baseline characteristics of patients with BR-PV and BR-A.

Variable	BR-PV (*n* = 88)	BR-A (*n* = 111)	Variable	BR-PV (*n* = 88)	BR-A (*n* = 111)
Sex (male/female)	51/37	55/56	Surgical procedures		
Age, years *	66 (39–83)	67 (42–83)	Pancreatoduodenectomy	74 (84%)	67 (60%)
Body mass index *	21.1 (15.4–43.6)	21.2 (11.6–30.7)	Distal pancreatectomy	1 (1%)	13 (12%)
Tumor location			Total pancreatectomy	7 (8%)	6 (5%)
Head/Uncinate	84 (95%)	83 (75%)	Operative time, min *	508 (308–960)	501 (193–808)
Body/Tail	4 (5%)	28 (25%)	Blood loss volume, mL *	1075 (258–6000)	1090 (80–9845)
CA19-9 at diagnosis, U/mL *	179 (1–2900)	150 (1–6340)	Operative PRBC transfusion	34 (41%)	30 (34%)
Chemotherapy			Vascular resection		
no	46 (52%)	48 (43%)	Any venous resection	72 (82%)	62 (56%)
FFX/GnP	26 (30%)	36 (32%)	Any arterial resection	5 (6%)	12 (14%)
GS	2 (2%)	27 (24%)	Celiac axis	0	5
GS + Radiation	14 (16%)	14 (13%)	Hepatic artery	4	8
Length of therapy, mo *	2.1 (1.1–6.6)	2.7 (0.2–12.9)	Splenic artery	1	0
Tumor size at operation, mm *	30 (9–100)	30 (10–100)	Both venous and arterial	5 (6%)	8 (9%)
CA19-9 at operation, U/mL *	93 (1–9869)	102 (1–7316)	Positive lymph nodes	54 (66%)	58 (67%)
in upfront surgery group	196 (1–9869)	321.5 (1–7316)	R0 margin status	59 (72%)	49 (56%)
in resection after NAT group	41 (1–1500)	34 (1–2690)	90-day operative mortality	1 (1%)	1 (1%)
CA19-9 normalized	17 (47%)	17 (27%)	Adjuvant chemotherapy	56 (68%)	75 (86%)
CA19-9 decrease rate ≥90%	7 (19%)	9 (19%)	Recurrent disease	42 (51%)	58 (67%)
Nutrition at operation			Vital status at last follow-up		
CONUT *	2 (0–11)	2 (0–11)	Alive, no evidence of recurrence	32 (36%)	34 (31%)
GPS *	0 (0–2)	0 (0–2)	Alive, with recurrence	7 (8%)	19 (17%)
mGPS *	0 (0–2)	0 (0–2)	Not alive	49 (56%)	58 (52%)
NLR *	2.4 (0.8–20.4)	2.5 (0.7–15)			
PLR *	129.2 (0.1–416.5)	83.0 (0.05–522.5)			
PNI *	46.0 (28.5–56.2)	44.5 (26.3–55.5)			
LMR *	3.6 (1.0–40.4)	3.8 (1.0–10.9)			
SII *	380.1 (0.2–2180.5)	482.8 (0.1–3669.2)			
CRP/Alb *	0.07 (0–2.0)	0.03 (0.002–2.2)			

* values are median (range). CA19-9, carbohydrate antigen 19-9; FFX, FOLFIRINOX; GnP, gemcitabine along with nab-paclitaxel; GS, gemcitabine along with S-1; CONUT, controlling nutritional status; GPS, Glasgow prognostic score; mGPS, modified Glasgow prognostic score; NLR, neutrophil/lymphocyte ratio; PLR, platelet/lymphocyte ratio; PNI, prognostic nutritional index; LMR, lymphocyte/monocyte ratio; SII, systemic immune inflammation index; CRP, C-reactive protein; Alb, albumin; PRBC, packed red blood cells.

**Table 2:** Baseline characteristics of patients with BR-PDAC who underwent resection.

Variable	BR-PV (*n* = 82)	BR-A (*n* = 87)
Sex (male/female)	47/35	44/43
Age, years *	65 (39–83)	67 (42–83)
Chemotherapy		
no	46 (56%)	48 (55%)
FFX/GnP	26 (32%)	36 (30%)
GS	2 (2%)	27 (31%)
GS + Radiation	14 (17%)	14 (16%)
Tumor size at operation, mm *	30 (9–100)	30 (10–100)
CA19-9 at operation, U/mL *	93 (1–9869)	102 (1–7316)
in upfront surgery group	196 (1–9869)	321.5 (1–7316)
in resection after NAT group	41 (1–1500)	34 (1–2690)
CA19-9 normalized	17 (21%)	17 (20%)
Surgical procedures		
Pancreatoduodenectomy	74 (90%)	67 (77%)
Distal pancreatectomy	1 (1%)	13 (15%)
Total pancreatectomy	7 (10%)	6 (7%)
Operative time, min *	508 (308–960)	501 (193–808)
Blood loss volume, mL *	1075 (258–6000)	1090 (80–9845)
Operative PRBC transfusion	34 (41%)	30 (34%)
Vascular resection		
Any venous resection	72 (88%)	62 (71%)
Any arterial resection	5 (6%)	12 (14%)
Celiac axis	0	5
Hepatic artery	4	8
Splenic artery	1	0
Both venous and arterial	5 (6%)	8 (9%)
Positive lymph nodes	54 (66%)	58 (67%)
R0 margin status	59 (72%)	49 (56%)
90-day operative mortality	1 (1%)	1 (1%)
Adjuvant chemotherapy	56 (68%)	75 (86%)

* values are median (range). CA19-9, carbohydrate antigen 19-9; FFX, FOLFIRINOX; GnP, gemcitabine along with nab-paclitaxel; GS, gemcitabine along with S-1; PRBC, packed red blood cells.

**Table 3:** Baseline characteristics of patients with BR-PDAC who underwent upfront surgery or NAT.

Variable	BR-PV (*n* = 88)		BR-A (*n* = 111)	
	UFS (*n* = 46)	NAT (*n* = 42)	UFS (*n* = 48)	NAT (*n* = 63)
Sex (male/female)	27/19	24/18	27/22	28/35
Age, years *	64 (39–83)	66 (40–81)	66 (42–83)	68 (45–82)
Body mass index *	20.1 (15.5–32.1)	21.4 (15.4–43.6)	20.9 (17.1–27.5)	21.4 (11.6–30.7)
Tumor location				
Head/Uncinate	44 (96%)	40 (95%)	38 (79%)	45 (71%)
Body/Tail	2 (4%)	2 (5%)	10 (21%)	18 (29%)
CA19-9 at diagnosis, U/mL *	N/A	178.5 (1–2900)	N/A	150 (1–6340)
CA19-9 at operation, U/mL *	196 (1–9869)	41 (1–5661)	321 (1–7316)	65 (1–5870)
Comorbidity (yes/no)				
Diabetes	20/26	11/26	23/25	13/34
History of other cancers	6/40	1/36	5/43	8/40
Pancreatitis	5/41	0/37	11/37	1/47
Hepatitis	2/44	1/36	4/44	3/45
Hypertension	11/35	12/25	13/35	19/29
Renal dysfunction	1/45	0/37	0/48	0/48
Nutrition at operation				
CONUT *	1.5 (0–10)	3 (0–11)	2 (0–11)	2 (0–10)
GPS *	0 (0–2)	0 (0–1)	0 (0–2)	0 (0–1)
mGPS *	0 (0–2)	0 (0–2)	0 (0–2)	0 (0–2)
NLR *	2.2 (1.1–20.4)	2.9 (0.8–8.6)	2.6 (1.0–9.7)	2.5 (0.7–15)
PLR *	97.7 (0.1–325.5)	166 (67.4–416.5)	104.2 (0.05–290)	184.9 (57.9–522.5)
PNI *	46.3 (29.5–56.2)	44.8 (28.5–52.5)	44.5 (26.3–55.5)	43 (32–51.5)
LMR *	4.1 (2.2–6.5)	3.1 (1.0–40.4)	5.4 (1.4–10.9)	3.5 (1–6.1)
SII *	300 (0.2–2180.5)	600 (168.4–1786.9)	376.9 (0.1–1944.4)	530.8 (95.1–3669.2)
CRP/Alb *	0.07 (0–2.0)	0.07 (0–3.3)	0.02 (0.002–1.0)	0.03 (0.002–2.2)

* Values are median (range). CA19-9, carbohydrate antigen 19-9; CONUT, controlling nutritional status; N/A, not available; GPS, Glasgow prognostic score; mGPS, modified Glasgow prognostic score; NLR, neutrophil/lymphocyte ratio; PLR, platelet/lymphocyte ratio; PNI, prognostic nutritional index; LMR, lymphocyte/monocyte ratio; SII, systemic immune inflammation index; CRP, C-reactive protein; Alb, albumin.

**Table 4:** Comparison of regimens in patients who underwent NAT.

	BR-PV (*n* = 42)						BR-A (*n* = 63)					
	*n*	MST (Months)	CA19-9 Normalized	Resection	R0 Rate	Evans Grade ≥IIb	*n*	MST (Months)	CA19-9 Normalized	Resection	R0 Rate	Evans Grade ≥IIb
FFX/GnP	26	32.9	47%	22 (85%)	86%	24%	29	35.4	40%	17 (59%)	71%	21%
FFX/GnP with RT	0						7	18.7	75%	4 (57%)	100%	0%
Old NAC	2	10	0%	2 (100%)	50%	0%	10	43.2	38%	9 (90%)	67%	0%
Old NAC with RT	14	20.6	50%	12 (86%)	100%	36%	17	19.7	20%	9 (53%)	100%	33%

Old NAC means neoadjuvant chemotherapy including gemcitabine, S-1, and GEM with S-1; FFX, FOLFIRINOX; GnP, gemcitabine along with nab-paclitaxel; RT, radiotherapy; NAC, neoadjuvant chemotherapy; MST, median survival time.

**Table 5:** Univariate and multivariate analyses of the clinical features of BR-PDAC patients who underwent resection after NAT.

	BR-PV Univariate			BR-PV Multivariate		BR-A Univariate			BR-A Multivariate	
Clinical Factor	No. Patients (*n* = 36)	HR (95% CI)	*p*	HR (95% CI)	*p*	No. Patients (*n* = 39)	HR (95% CI)	*p*	HR (95% CI)	*p*
Radiation in NAT	12	0.96 (0.23–4.04)	0.955			12	1.37 (0.38–4.87)	0.63		
CA19-9 Before NAT >192 U/mL	17	2.37 (0.58–9.64)	0.23			19	0.41 (0.22–1.48)	0.175		
Preoperative CA19-9 >34 U/mL	17	1.05 (0.26–4.27)	0.945			19	3.90 (0.97–15.72)	0.056		
Tumor marker normalization	20	0.16 (0.031–0.82)	0.028 *	0.28 (0.05–1.71)	0.168	16	0.10 (0.01–0.83)	0.033 *	0.15 (0.01–1.57)	0.064
Preoperative Alb >3.8 g/dL	14	0.55 (0.14–2.24)	0.404			17	0.30 (0.06–1.46)	0.137		
Preoperative CONUT score, >4	5	3.78 (0.92–15.56)	0.065			9	2.48 (0.66–9.34)	0.179		
Preoperative GPS 0	16	0.15 (0.03–0.79)	0.025 *	0.50 (0.05–4.68)	0.547	10	0.52 (0.07–3.78)	0.52		
Preoperative mGPS 0	14	0.25 (0.05–1.28)	0.095			22	0.26 (0.06–1.08)	0.064		
Preoperative NLR >2.52	16	3.17 (0.39–25.86)	0.281			18	1.47 (0.39–5.51)	0.571		
Preoperative PLR >184	11	0.97 (0.23–4.10)	0.972			19	4.15 (0.85–20.26)	0.079		
Preoperative PNI >42.5	16	0.15 (0.03–0.76)	0.022 *	0.32 (0.03–2.98)	0.316	21	0.13 (0.03–0.65)	0.013 *	0.15 (0.02–0.85)	0.014 *
Preoperative LMR >3.50	9	0.19 (0.02–1.72)	0.141			14	0.42 (0.11–1.64)	0.214		
Preoperative SII >512	15	0.89 (0.21–3.74)	0.873			19	4.34 (0.84–22.49)	0.08		
Preoperative CRP/Alb >0.062	12	2.20 (0.43–11.16)	0.341			10	1.75 (0.43–7.08)	0.431		
Preoperative diabetes	11	0.78 (0.18–3.33)	0.739			13	0.91 (0.23–3.55)	0.9		
Preoperative treatment period >60 day	19	1.23 (0.15–10.09)	0.844			29	0.46 (0.12–1.67)	0.237		
Preoperative treatment period >90 day	7	1.72 (0.34–8.67)	0.509			14	1.07 (0.28–4.18)	0.918		
Operative time >560 min	7	3.12 (0.74–13.14)	0.121			20	1.73 (0.49–6.09)	0.396		
Intraoperative blood loss >830 ml	21	1.06 (0.25–4.45)	0.941			20	7.42 (1.53–36.1)	0.013 *	2.23 (0.37–13.35)	0.358

CA19-9, carbohydrate antigen 19-9; NAT, neoadjuvant treatment; Alb, albumin; CONUT, controlling nutritional status; GPS, Glasgow prognostic score; mGPS, modified Glasgow prognostic score; NLR, neutrophil/lymphocyte ratio; PLR, platelet/lymphocyte ratio; PNI, prognostic nutritional index; LMR, lymphocyte/monocyte ratio; SII, systemic immune inflammation index; CRP, C-reactive protein; Alb, albumin; * *p* < 0.05.

**Table 6:** Resectability criteria proposed by the JPS.

Resectability	SubClass	Detail
1. Resectable (R)		No contact of the tumor with the SMV/PV.
		Abutment/encasement of the SMV/PV of <180° circumference without occlusion (termed R-PV).
		No contact with any major artery (CA, SMA, or CHA).
2. Borderline resectable (BR)	BR-PV	
		Tumor abutment/encasement or occlusion of the SMV/PV of ≥180°.
		No arterial tumor abutment/encasement (CA, SMA, or CHA).
	BR-A	
		Tumor abutment/encasement of the SMA or CA of <180° without irregularity in the contour of the artery.
		Tumor abutment/encasement of the CHA without irregularity in the contour of the PHA or CA.
3. Unresectable (UR)	UR-LA (Locally advance)	
		Tumor abutment/encasement of the SMA or CA of ≥180°.
		Tumor abutment/encasement of the CHA and extension of abutment/encasement to the PHA or CA.
		Tumor abutment/encasement of the aorta.
	UR-M (Metastasis)	
		Distant metastases, including metastases to lymph nodes beyond regional lymph nodes.

JPS, Japan Pancreas Society; SMV, superior mesenteric vein; PV, portal vein; CA, celiac artery; SMA, superior mesenteric artery; CHA, common hepatic artery; PHA, proper hepatic artery.
